# MiR-200c-3p Regulates DUSP1/MAPK Pathway in the Nonalcoholic Fatty Liver After Laparoscopic Sleeve Gastrectomy

**DOI:** 10.3389/fendo.2022.792439

**Published:** 2022-03-01

**Authors:** Tao-tao Zhang, Yong Wang, Xiang-wen Zhang, Ke-yu Yang, Xiu-qin Miao, Guo-hua Zhao

**Affiliations:** ^1^ General Surgery, Dalian Municipal Central Hospital, Dalian, China; ^2^ General Surgery, Fourth Affiliated Hospital of China Medical University, Shenyang, China

**Keywords:** NAFLD, sleeve gastrectomy, miR-200c-3p, DUSP1, MAPKs

## Abstract

**Aim:**

Non-alcoholic fatty liver disease (NAFLD) is a health burden worldwide, which is closely related to obesity. The effect of sleeve gastrectomy (SG) on NAFLD is efficient, and the underlying mechanism remains unknown. Our study sought to investigate the mechanism of dual-specificity protein phosphatase 1 (DUSP1) expression regulation following the SG procedure in NAFLD patients and C57BL/6J mice *via* miR-200c-3p.

**Methods:**

The serum was extracted from NAFLD patients who underwent laparoscopic sleeve gastrectomy (LSG) and volunteers. Next, the correlation between miR-200c-3p and DUSP1 was identified *in vitro*. NAFLD mice were modelled by high-fat diets (HFD). The hepatic tissue expression levels of miR-200c-3p, DUSP1, phospho-extracellular regulated protein kinases1/2 (p-ERK1/2), phospho -p38 mitogen-activated protein kinases (p-p38), and phospho-c-Jun N-terminal kinases (p-JNK) induced by SG procedure were evaluated.

**Results:**

The SG procedure contributed to significant weight loss, reduced lipids in NAFLD patients and mice. The increased expression level of miR-200c-3p and reduced expression of DUSP1 were observed in NAFLD patients and mice (p<0.05). The reduced expression levels of miR-200c-3p and increased expression of DUSP1 were observed in patients and mice with NAFLD who underwent SG procedure. DUSP1 is a potential target of miR-200c-3p.

**Conclusions:**

A novel mechanism was identified in which miR-200c-3p regulates the MAPK-dependent signals that are linked to the promotion of hepatosteatosis *via* DUSP1 after sleeve gastrectomy. The findings suggested that miR-200c-3p should be further explored as a potential target for the treatments of NAFLD.

## Introduction

NAFLD is the most common cause of liver disease worldwide, considered the hepatic manifestation of metabolic syndrome associated with obesity, insulin resistance (IR), dyslipidemia, diabetes, and heredity (1). NAFLD encompasses a spectrum of diseases includes nonalcoholic hepatic steatosis, nonalcoholic steatohepatitis (NASH), cirrhosis, and hepatocellular carcinoma(HCC) (2, 3). Currently, nearly 1 billion people worldwide are affected by NAFLD due to the dramatic increase in obesity, with the incidence of NAFLD in the Asian population reaching about 27% (4). The most recent national data showed that 16.4% of Chinese adults had obesity (BMI 28.0 kg/m² or higher) and another 34.3% were overweight (24.0–27.9 kg/m²) (5).

Overweight and obesity were the sixth leading risk factor for death and disability (6). Obesity is an independent risk factor for NAFLD, and weight loss is the only safe and effective treatment for NAFLD (7, 8). A recent randomized controlled study found that a low-calorie diet sustained for 3 months could significantly reduce body weight (4.5%) and improve liver enzymes, nevertheless, without significant changes in liver adipose degree (9). Physical exercise is recommended as a routine treatment for NAFLD (10). Metformin, sodium-glucose transporter 2 (SGLT2) inhibitors, lipase inhibitors, and Glucagon-like peptide 1 (GLP 1) receptor stimulants all have been reported weight reduction effects (11–13). However, it is difficult for most obese patients to achieve and maintain an ideal state of body mass after lifestyle intervention and medical treatment, and surgical treatment should be considered.

Bariatric surgery is the most effective way to lose weight in morbid obesity. In patients with biopsy-proven NAFLD/NASH, bariatric procedures are similarly effective in improving liver function (1). Nevertheless, fatal hyper-ammonemic encephalopathy encompassing genetic and non-genetic causes was reported after laparoscopic Roux-Y gastric bypass (LRYGB) (14, 15). Absorption-restricted surgery may cause the displacement of intestinal flora and the activation of the inflammatory system, which causes endotoxin damage to the liver, and now there are some cases of liver function deterioration (16–18). No exacerbation of NAFLD after laparoscopic sleeve gastrectomy (LSG) has been reported. LSG is a bariatric surgical technique that can result in considerable weight loss with negligible complications (19).

DUSP1, also known as MKP 1(mitogen-activated protein kinase phosphatase 1, MKP 1), localizes to the nucleus, can be activated by stress or misuse induction, and can selectively inactivate the MAPK signaling pathway (20). DUSP1 was downregulated 2.04 FC (fold change, FC) between Definite NASH and Not NAFLD, 1.94 FC between Definite NASH and Borderline (21). MAPK signaling pathway is involved in the two “hits” of NAFLD.

MicroRNAs are small, non-coding RNAs that are essential post-transcriptional regulators of gene expression. By binding to the 3’ untranslated region (3’UTR) of their target genes, they globally repress gene expression (22). The gene encoding miRNA is located in the nucleus, and the miRNA is transcribed into pre-miRNA with the action of RNA polymerase pol II. Dorsha endonuclease further transformed it into an intermediate pre-miRNA with a stem-loop structure of about 60 bases, which is then transported into the cytoplasm. After the action of Dicer endonuclease, the intermediate pre-miRNA formed an incomplete pairing miRNA-miRNA double-stranded complex. It also plays a role in gene expression regulation through the formation of a nucleic acid-protein complex (miRNP) (23–25). A previous study reported that miR-200c-3p was up-regulated in NAFLD of rats (26). However, there was no in-depth investigation on miR-200c-3p before and after bariatric surgery.

This study aims to investigate the mechanism of dual-specificity protein phosphatase 1 (DUSP1) expression regulation following the sleeve gastrectomy (SG) procedure in NAFLD patients and C57BL/6J mice *via* miR-200c-3p. In our study, we demonstrated that miR-200c-3p regulates DUSP1 expression in the HepG2 cell line, and its expression was increased in NAFLD patients and mice. Our results suggested that the SG procedure could significantly ameliorate the NAFLD compared with food restriction, miR-200c-3p expression level was decreased after the SG procedure. Decreased expression of miR-200c-3p increased hepatic DUSP1, decreased MAPK activity, which plays a protective role in the development and progression of NAFLD.

## Materials and Methods

### Ethics Statement

All the procedures within this study were performed following the Helsinki declaration of 1975, 1983 revision. All human and animal studies were approved by the committee of our center (YN2020-028-04). All methods were carried out following the relevant guidelines and regulations.

### Human Subjects Experimentation

The study was a retrospectively observational design, which consisted of 7 NAFLD patients with BMI> 27.5 kg/m^2^ who underwent LSG and five healthy volunteers with no NAFLD admitted to our center from September 2018 to September 2019. Inclusion criteria: (a) the age of patients and volunteers was at least 18 to 60 years old (including both ends) at the time of signing the informed consent; (b) meeting the LSG surgical indications and successfully performing LSG surgery; (c) being able to follow the case manager’s requirements for a regular diet and out-patient visit follow-up;(d) being approved by the ethics committee; (e) the diagnostic criteria for NAFLD and the criteria for ultrasound evaluation were following the guidelines for the prevention and treatment of NAFLD (27). Exclusion criteria: (a) patients underwent LSG procedure but not complicated with NAFLD; (b) failure to follow up regularly after surgery; (c) alcoholic liver disease, patients who drink alcohol equivalent to the amount of ethanol >140g per week for men or >70g for women; (d) viral hepatitis, drug-induced liver disease, total parenteral nutrition, hepatolenticular degeneration, autoimmune liver disease and other specific diseases that can lead to NAFLD; (e) liver function damages caused by other causes.

The clinical biochemical and physical indicators of the patients were followed up, the expression of miR-200c-3p in peripheral sera was determined by quantitative real-time transcription-polymerase chain reaction (qRT-PCR) and the expression of DUSP1 in the blood was determined by enzyme-linked immunosorbent assay (Elisa). BMI was calculated as weight (in kilograms) divided by height (in meters) squared. Total serum cholesterol and triglycerides, hepatic enzyme, and other routine laboratory tests were measured as previously. Patients were requested to withhold alcohol and caffeine for at least 12 h before the collecting of blood samples. The blood samples were collected one day before the surgery, the 1st month, 3rd month, and 6th month after surgery, respectively.

### Elisa

ELISA kit (SEC902Hu, USCN KIT INC) was pre-coated with an antibody specific to the DUSP1 antibody. Standards or samples were then added to the appropriate microplate wells with a biotin-conjugated antibody specific to DUSP1. Next, Avidin conjugated to Horseradish Peroxidase (HRP) was added to each microplate well and incubated. Followed by incubation at room temperature. After tetramethylbenzidine (TMB) substrate solution was added, only those wells that contain DUSP1, biotin-conjugated antibody, and enzyme-conjugated. After the termination of the reaction, the uncoupled conjugate was washed away. Avidin will exhibit a color change to blue and turn yellow after the addition of an acidic stop solution. The density of yellow and the content of DUSP1 in the samples were in proportion to the bottom of the kit. Then, the concentration of DUSP1 in the samples was then determined by comparing the optical density (OD) of the samples to the standard curve.

### Animal Model of NAFLD

Seven-week-old male C57BL/6J mice (*n*=30) (approx 22gm body weight) were obtained from Liaoning Changsheng biotechnology company limited and maintained on a standard chow diet ad libitum and a standard 12 h:12 h light/dark cycle until eight weeks of age. Mice at this age were then given a high-fat diet (Research Diets D12451, 45 kcal% saturated fat, n=22), and regular chow (5% fat, 53% carbohydrate, and 23% protein) was given to control rats (*n* = 8) for 12 weeks. One mouse was randomly selected from the control and NAFLD groups respectively, and the hepatic tissue was harvested and stained with Hematoxylin and eosin (HE) and oil red O methods. After confirming the successful modeling of NAFLD, the NAFLD group was then randomly divided into three groups, followed by the NAFLD+SG group (*n*=7), NAFLD+Food restriction group (FR, n=7) and NAFLD+Sham surgery group (*n*=7). The mice in the CON group were maintained on standard chow for 18 weeks. The weight and food intake of the mice in the different groups were documented weekly.

### Serum Biochemical Assays

At the end of the experiment, all mice were sacrificed, peripheral blood was collected from the vein of the inner canthus. Triglycerides (TG), total cholesterol (TC), alanine aminotransferase (ALT), aspartate aminotransferase (AST), high-density lipoprotein cholesterol (HDL-c) and low-density lipoprotein cholesterin (LDL-c) were assayed.

### SG Surgery

Four percent of isoflurane was used to induce anesthesia of operated mice, and 2% isoflurane was used to maintain anesthesia. Seventy to eighty percent of the lateral stomach was excised, leaving a tubular gastric remnant in continuity with the esophagus superiorly and the pylorus and duodenum inferiorly. The NAFLD+SHAM group involved analogous isolation of the stomach followed by manually applying pressure with blunt forceps along a vertical line between the esophageal sphincter and the pylorus. After the surgery, mice were maintained on a liquid diet (ENSURE) during the 7-day recovery period. The HFD diet in the NAFLD+SG group was weighed once a day to calculate the average daily intake of each mouse. The NAFLD+FR group was kept the same designated diet as the NAFLD+SG group after the 7-day recovery period. Both NAFLD+SG and NAFLD+SHAM groups received gentamicin for seven days after surgery.

### RNA Preparation and Quantitative Real-Time PCR (qRT-PCR)

Human peripheral blood serum was centrifuged at 3000 RPM (Revolution Per Minute) for 5 minutes and refrigerated at -80°C, total RNA was extracted using TRIpure (RP1001, BioTeke, Beijing) following the manufacturer’s instructions. Mice hepatic tissue and HepG2 cells were lysed, and total RNA was extracted using TRIpure (BioTeke, Beijing). For microRNA analysis, miRNA-specific cDNA was generated with Super M-MLV reverse transcriptase (BioTeke, Beijing). Primer sequences were synthesized in GenScript Biotechnology (**Table 1**) and followed by qRT-PCR using SYBR Green master mix (Solarbio, Beijing) in Exicycler 96 (BIONEER, Korea). Relative gene expression levels were calculated by the 2 ^-ΔΔCT^ method.

**Table 1:** Primer sequences for RT-qPCR.

NAME^*^	Sequence(5’→ 3’)
U6 F	CGCAAGGATGACACGCAAAT
U6 R	GCAGGGTCCGAGGTATTC
mmu-miR-200c-3p F	GCCGGGTAATGATGGAGT
mmu-miR-200c-3p R	GCAGGGTCCGAGGTATTC
hsa-miR-200c-3p F	GCCGGGTAATGATGGAGT
hsa-miR-200c-3p R	GCAGGGTCCGAGGTATTC
DUSP1 mRNA F	GTGCCTATCACGCTTCTCG
DUSP1 mRNA R	CCTCCACAGGGATGCTCTT
β-actin F	CTGTGCCCATCTACGAGGGCTAT
β-actin R	TTTGATGTCACGCACGATTTCC

*Abbreviations: F, forward primer; R, reverse primer.

### MRNA Extraction and Quantitative Analysis

RNA was isolated using TRIZOL (RP1001, BioTeke, Beijing) from the HepG2 cells and hepatic tissue of C57BL/6J. RT-PCR was performed using BeyoRT II M-MLV reverse transcriptase (Beyotime, Shanghai, China) and a custom-made DUSP1 primer (GenScript Biotechnology, **Table 1**). The data were normalized to β-actin mRNA. RT-PCR was conducted in a reaction volume of 20 μl.

### Western Blotting

The hepatic tissue was rapidly removed after the mice’s sacrifices, immediately frozen in liquid nitrogen. The hepatic tissue of mice and HepG2 cells were lysed in Whole-Cell Lysis Assay (Solarbio, China) for 5 min on ice and centrifuged (12 000 rpm, 10 min, and 4°C). Protein concentrations were measured using a bicinchoninic acid assay kit protein assay kit (Wanleibio, China) according to the manufacturers’ instructions. The diluted protein samples were mixed with loading buffer (5×; Wanleibio, China). Then the samples were boiled for 5 min at 95˚C. Proteins (40ug/lane) were separated by 10% SDS-polyacrylamide gel electrophoresis and transferred to the PVDF membrane (80V, 1.5h). The membranes were blocked by a solution of 5% non-fat dried milk or albumin from bovine serum (BSA) in Tris-buffered saline with Tween (TBST) for 1h at room temperature. The membranes were then incubated at 4 overnight with primary antibodies for DUSP1 antibody (the dilution ratios 1:1000, Abclonal, China), β-actin antibody (the dilution ratios 1:400, Wanleibio, China), p-ERK1/2(the dilution ratios 1:400, Wanleibio, China), p-p38(the dilution ratios 1:500, Wanleibio, China), and p-JNK(the dilution ratios 1:500, Wanleibio, China). After washing with the TBST, membranes were incubated with IgG-horseradish peroxidase-conjugated sheep anti-rabbit secondary antibodies (the dilution ratios 1:5000, Wanleibio, China) for 45 min at 37˚C. Membranes were then washed six times with TBST and the chemiluminescent signals were detected with enhanced chemiluminescent (ECL) luminous fluid (Wanleibio, China) using the Gel-Pro-Analyzer System (WD-9413B, China).

### Bioinformatics Analysis

The bioinformatics analysis for identifying target genes and microRNAs was done using StarBase and TargetScan software tools.

### Transcriptional Activity by Luciferase Reporter Assays

Briefly, the 293T cells purchased from Shanghai Zhong Qiao Xin Zhou Biotechnology were cultured in DMEM (Dulbecco’s modified eagle medium, Gibico) containing 10% fetal calf serum at 37°C under 95% humidity and 5% CO_2_. Subsequently, the pmirGLO-DUSP1-3’UTR-wt (Genscript, Nanjing) or pmirGLO-DUSP1-3’UTR-mut vector (Genscript, Nanjing), along with the miR-200c mimic or the mimic-control, were transfected with Lipofectamine ^®^ 2000 into 293T cells. Following 48 h, the cells were then lysed using passive lysis buffer, and the luciferase activity was measured using a Dual-Luciferase Reporter Gene Assay Kit (KeyGEN BioTECH, China). Renilla luciferase activity was used for the normalization of the firefly luciferase activity. The luciferase enzyme activity was presented as fold-change relative to the vehicle control.

### Cell Culture and Transfection

The HepG2 cells were seeded in DMEM (Dulbecco’s modified eagle medium, Gibico) medium containing 10% fetal bovine serum for 24 h before transfection and transfected with miR-200c mimics (Jintuosi, China), miR-200c inhibitor (Jintuosi, China), and NC (Jintuosi, China) for 44h. The groups were as follows: A: Non-transfected group (HepG2); B: Negative mimic control group (HepG2+mimic-NC); C: Transfected group with mimic (HepG2+ miR-200c-3p mimic); D: H_2_O_2_ intervention in the mimic group (HepG2+miR-200c-3p mimic+H_2_O_2_); E: Negative control group (HepG2+inhibitor NC); F: Inhibitor transfection group (HepG2+miR-200c-3p inhibitor); G: H_2_O_2_ intervention group (HepG2+ miR-200c-3p inhibitor+H_2_O_2_). The cells were cultured in an incubator at 37°C with 5% CO_2_ until the cells adhered to the wall. Transfection could be performed when cell density was 70%. Preparations were as follows: Solution 1: Optimized solution 100μ L + LIPO 2000 6μ L, fully mixed and placed at room temperature for 5 min; Solution 2: Optimized solution 100μ L + fragment 100pmol, the same as solution 1. After mixing the two solutions thoroughly and evenly, let them stand for 20 minutes at room temperature. After the above solution was slowly added to the cells, gently shaken continuously, and then the cells were cultured in an electrothermal constant temperature incubator (37°C, 5%CO_2_). The Group D and G cells were treated with H_2_O_2_ (100 uM) for four hours. Cells in different groups were harvested after 48 h of transfections for the subsequent detections.

### Haematoxylin-Eosin Staining

Haematoxylin-eosin staining (HE) was conducted according to standard frozen section protocols. Briefly, after the sections were embedded, ten µm longitudinal sections were rinsed three times in distilled water for 5 mins respectively, and then stained with hematoxylin solution for 5 mins followed by 3 secs in 1% acid ethanol (1% HCl in 70% ethanol), then the sections were washed with tap water for 10 mins. After that, the sections were rinsed in distilled water for 2 mins. Then the sections were stained with eosin solution for 3mins and followed by dehydration with graded alcohol. Next, routine dehydration, clearing, and mounting were performed: 95% ethanol (I) for 5 mins, 95% ethanol (II) for 5 mins, xylene (I) for 10 mins, and xylene (II) for 10 mins. Finally, the sections were mounted by neutral resin, which was then observed and photographed using an Olympus BX53 fluorescence microscope (Tokyo, Japan).

### Lipid Deposition Analysis by Oil Red O Staining

Histological visualization of lipid deposition in the mice’s hepatic tissue was carried out using oil red O staining. Briefly, the mice liver sections were first incubated in distilled water for 2 mins, then 60% propylene glycol for 2 mins, and then in oil red O solution for 5 mins. The sections were soaked in the 60% propylene glycol until the interstitial tissue was colorless. After being rinsed for 2 mins with distilled water, the slides were incubated at 37°C with Hematoxylin for 1 min, then mounted with gelatin mounting medium.

### Statistical Analysis

The continuous variables were shown as the mean and standard deviation; the categorical variables were shown as the number and percentage. Each experiment was performed in at least triplicate. Paired t Student tests were used to compare baseline data with pre-and postoperative ones. One-way analysis of variance (ANOVA) was used to compare the means of multiple samples in a group design. Wilcoxon tests were used for non-normal distributions. Statistical analysis was performed using SPSS version 23.0 (IBM Corporation, New Orchard Road Armonk, NY 10504. Produced in the United States of America). The graphics were performed using Graph Pad Prism 8.0 (GraphPad Software, Inc., La Jolla, CA, USA). A p value<0.05 was considered statistically significant.

## Results

### LSG Induced Weight Loss and Amelioration of NAFLD

**Figure 1:**
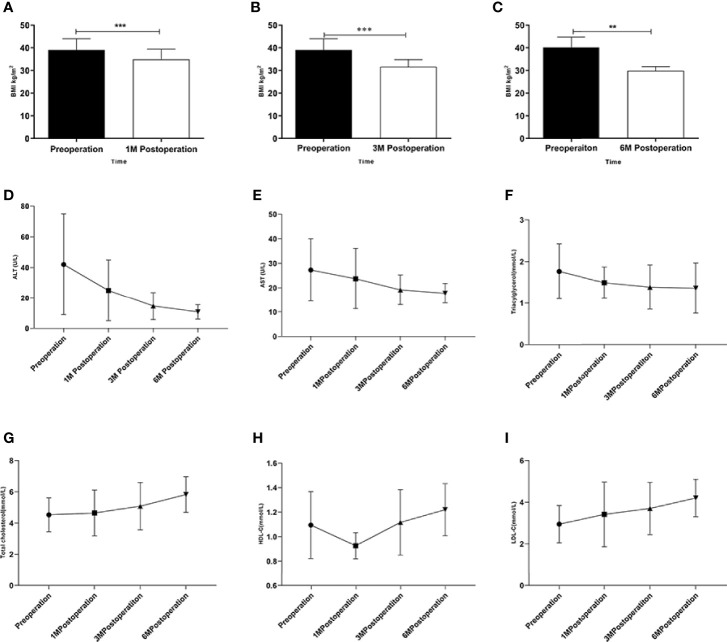
Changes in obese patients combined with NAFLD underwent LSG. **(A–C)** Changes in BMI after the LSG procedure during the follow-up at four time points; **(D–I)** Changes in ALT、AST、TC、TG、HDL-C and LDL-C after LSG procedure during the follow-up at four time points. **p < 0.01; ***p < 0.001. Measurement data were expressed as mean ± standard deviation and compared by paired t-test or one-way ANOVA.

There was no death or serious complication during the follow-up period. According to the general hepatic ultrasound results, only 1 patient showed no improvement during the follow-up period. Changes in BMI were plotted over time from surgery in obese patients combined with NAFLD (**Figures 1A**–**C**). ALT, AST, TG showed a downward trend (**Figures 1D**–**F**) and TC, HDL-C, LDL-C showed an upward trend after the operation (**Figures 1G**–**I**).

### LSG Downregulated miR-200c-3p and Upregulated DUSP1

**Figure 2:**
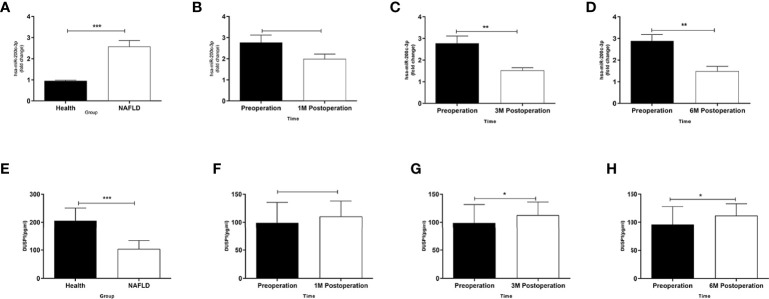
Different expressions of miR-200c-3p and DUSP1 in serum of patients. **(A)** MiR-200c-3p expressions in serums of NAFLD patients and healthy volunteers determined by RT-PCR (normalized to U6). **(B–D)** Changes in miR-200c-3p in serum of obese patients combined with NAFLD after LSG procedure during follow-up at four time points. **(E)** DUSP1 expressions in serums of NAFLD patients and healthy volunteers determined by ELISA. **(F–H)** Changes in DUSP1 in serum of obese patients combined with NAFLD after LSG procedure during follow-up at four time points. *p < 0.05; **p < 0.01; ***p < 0.001. Measurement data were expressed as mean ± standard deviation and compared by paired t-test.

The expression level of miR-200c-3p in the NAFLD group was upregulated and DUSP1 downregulated compared with healthy volunteers (**Figures 2A, E**). The expression level of miR-200c-3p was downregulated and DUSP1 was upregulated at the 3rd month postoperation with statistical significance (**Figures 2C, G**), the trend was more obvious at the 6th month (**Figures 2D, H**).

### MiR-200c-3p Bonded With DUSP1 3’UTR to Inactive DUSP1 Expression

**Figure 3:**
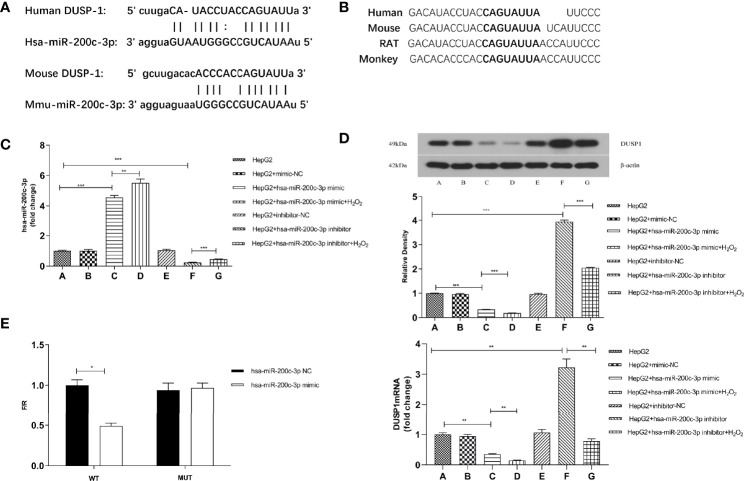
DUSP1 expression was directly regulated by miR-200c-3p. **(A)** Bioinformatics prediction of the binding sites between miR-200c-3p and DUSP1. **(B)** The predicted 8-mer binding region (black bold sequence) of miR-200c-3p. **(C)** miR-200c-3p expressions in different groups of HepG2 cell line. **(D)** The DUSP1 mRNA (normalized β-actin) and protein expressions (normalized to β-actin) in different groups of HepG2 cell line. **(E)** The luciferase activity of WT-DUSP1-3’UTR and MUT-DUSP1-3’UTR detected by dual-luciferase reporter assay. *p < 0.05; **p < 0.01; ***p < 0.001. Measurement data were expressed as mean ± standard deviation and compared by paired t-test.

We observed 3’UTR of DUSP1 mRNA showing a complementary site for the seed region of miR-200c-3p using miRNA target prediction programs (StarBase and TargetScann, **Figures 3A, B**). Western Blotting and RT-qPCR were used to confirm the role of miR-200c-3p in regulating DUSP1 expression in the HepG2 cell line. The miR-200c-3p expressions in the different groups were determined with RT-qPCR (**Figure 3C**). The DUSP1 mRNA and protein expression levels were lower in the miR-200c-3p mimic group and higher in the inhibitor group than in the CON group (**Figure 3D**).

H_2_O_2_ (100uM) was used in the miR-200c-3p mimic and inhibitor groups for stimulating oxidative damage. The results showed that the DUSP1 mRNA and protein expression levels were lower in the H_2_O_2_ groups and the miR-200c-3p were upregulated (**Figure 3D**).

The predicted region, containing the wildtype or mutant seed sequence of miR-200c-3p in the 3’UTR of DUSP1, was cloned into the luciferase reporter plasmid. The 293T cells transfected with the miR-200c-3p mimic and the DUSP1-3’UTR had a lower luciferase intensity, but the mutant reporter-transfected group did not, thus indicating that DUSP1 was a direct target of miR-200c-3p (**Figure 3E**).

### SG Induced Weight Loss, Amelioration of Lipid Panel and Liver Functions, Downregulated miR-200c-3p and Upregulated DUSP1 mRNA and Protein Expression in C57BL/6J Mice

**Figure 4:**
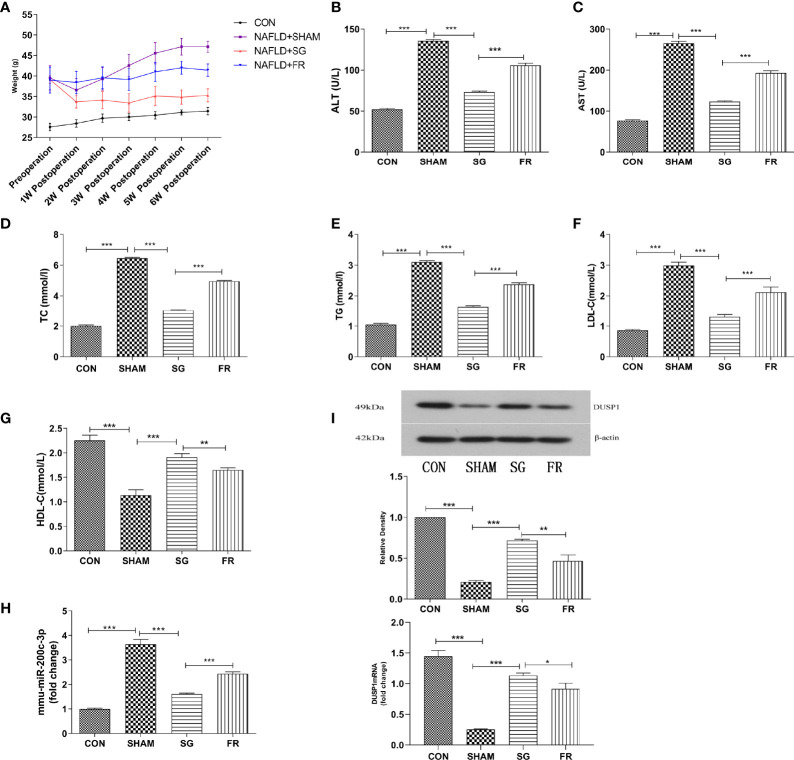
Weight loss, amelioration of lipid panel and liver functions, downregulated miR-200c-3p, and upregulated DUSP1 mRNA and protein expression in C57BL/6J mice. **(A)** Changes in body weight of C57BL/6J mice in different groups. **(B–G)** Different expressions of ALT、AST、TC、TG、LDL-C、HDL-C in different groups of C57BL/6J mice after SG procedure. **(H)** Different expressions of mmu-miR-200c-3p in different groups of C57BL/6J mice. **(I)** Different expressions of DUSP1 mRNA and protein in different groups of C57BL/6J mice. CON, Control group; SHAM, NAFLD+SHAM group; SG, NAFLD+SG group; FR, NAFLD+F R group. *p < 0.05; **p < 0.01; ***p < 0.001. Measurement data were expressed as mean ± standard deviation and compared by one-way ANOVA.

The bodyweight of C57BL/6J mice in different groups was measured weekly following SG procedures (**Figure 4A**). At the 6th week postoperatively, the bodyweight in the NAFLD+SG group was significantly decreased compared with the NAFLD+FR group and NAFLD+ SHAM group (**Figure 4A**). Consistent with the changes in body weight, lipid panel, and hepatic enzymology analysis also demonstrated that TC、TG、LDL-C、ALT、AST of the NAFLD+SG group were greatly lowered compared with the NAFLD+SHAM and NAFLD+FR group (**Figures 4B–F**). The HDL-C of the NAFLD+SG group was statistically increased compared with NAFLD+SHAM and NAFLD+FR group (**Figure 4G**). Our results demonstrated that miR-200c-3p were distinctly increased in the NAFLD+SHAM group, compared with the CON group (**Figure 4H**). The SG procedure decreased the miR-200c-3p expression (**Figure 4H**). On the contrary, the expression level of DUSP1 mRNA and protein were upregulated in the NAFLD+SG group compared with NAFLD+SHAM and NAFLD+FR group (**Figure 4I**).

### SG Causes the DUSP1 Mediated Amelioration of NAFLD

**Figure 5:**
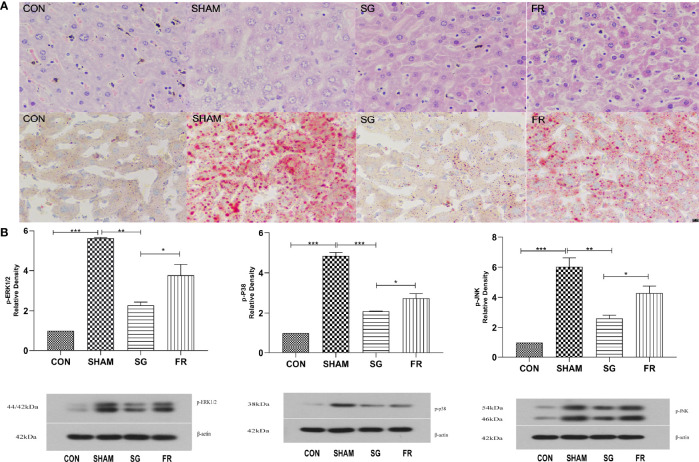
Changes of liver pathology and MAPKs in C57BL/6J mice. **(A)** Lipid accumulations in different groups of C57BL/6J mice after SG procedure determined with HE staining and Oil Red O staining (× 400). **(B)** Expressions of p-ERK1/2, p-p38, and p-JNK in different groups of C57BL/6J mice. *p < 0.05; **p < 0.01; ***p < 0.001. Measurement data were expressed as mean ± standard deviation and compared by paired t-test.

HE staining and oil red O staining results were described and diagnosed by two pathologists without special morphometric analysis. Results of the oil red-O dyed-tissues and HE staining showed that the fat deposition in the NAFLD group was greatly enhanced compared with the CON group, but the SG procedure significantly repressed lipid deposition in the NAFLD+SG group compared with NAFLD+SHAM and NAFLD+FR group (**Figure 5A**). DUSP1 specifically dephosphorylates the members of the MAPK kinase family, including ERK1/2, p38, and JNK. At the sixth week postoperatively, we demonstrated that p-ERK1/2, p-p38 and p-JNK were significantly downregulated in the NAFLD+SG group compared with the NAFLD+SHAM and NAFLD+FR group (**Figure 5B**).

## Discussion

Currently, weight loss is the only approved safe and effective treatment for NAFLD (28, 29). Bariatric surgery is superior to lifestyle modifications for treating patients with morbid obesity (30). However, controversy still exists concerning the impacts of bariatric surgery in patients with NAFLD (31). LSG can result in a considerable weight loss with negligible complications (19); nevertheless, the mechanism of SG in NAFLD remains unclear.

Ultrasonography is the most commonly used imaging examination method for the evaluation of NAFLD (32). Previous literature reported that only 6.3% still had some grade of steatosis one year after LSG (33). Results of general ultrasonography demonstrated that only one patient (14.3%) showed no improvement during the follow-up period in our study, which may be due to the insufficient follow-up time. The clinical follow-up data demonstrated that LSG could alleviate NAFLD.

The expression level of miR-200c-3p was significantly increased in the hepatic tissue of NAFLD rats (26, 34). Lin et al. (35) reported that miR-200c-3p showed an increasing trend in diabetic DB/DB mice liver tissue using miRNA array. Ramachan-Dran et al. (36) reported that miR-200c-3p promotes the progression of hepatitis C fibrosis by regulating the cellular Src (cellular sarcoma gene, cSrc) kinase signal cascade reaction through fas-associated phosphatase 1 (FAP1). In the present study, we demonstrated that the expression of miR-200c-3p in the serological specimens of patients with NAFLD was markedly upregulated in NAFLD patients, and then distinctly decreased following the LSG procedure, with the remission of NAFLD (**Figures 2A**–**H**).

DUSP1, a nucleus localized MKP, is a major negative regulator of the MAPK pathway and participated in maintaining homeostasis of glucose metabolism and energy balance in peripheral tissues (37, 38). Microarray analysis showed that the DUSP1 of hepatic tissue was downregulated in NAFLD compared with volunteers without NAFLD (21). In cardiomyocytes treated with high glucose, the inhibition of miR-200c-3p led to the overexpression of DUSP1 and the low expression of p-ERK1/2, p-P38, and p-JNK, thus attenuating the cardiac hypertrophy (39). Our study demonstrates that LSG could downregulate the serum expression of miR-200c-3p and reduce the degradation of DUSP1 (**Figures 2A**–**H**) at the 3rd month postoperation in patients with NAFLD.

As a result of the *in vivo* studies being complex and accompanied by other factors, we extended these observations from NAFLD patients to HepG2 and 293T cell lines *in vitro*. The DUSP1 mRNA and protein expression levels were lower in the miR-200c-3p mimic group than in the CON group (**Figure 3D**). Besides, the DUSP1 mRNA and protein expression levels were higher in the miR-200c-3p inhibitor group than in the CON group (**Figure 3D**). H_2_O_2_ can induce large amounts of reactive oxygen species (ROS), which can initiate the oxidative stress response, 0and was widely used to construct cell models of oxidative stress (40). It has been reported that H_2_O_2_ can induce the DUSP1 expression in the breast cell line MCF-7 (41). However, in our research, HepG2 cells were induced by 100uM H_2_O_2_ for 4 hours, the DUSP1 mRNA and protein expression levels were downregulated in the H_2_O_2_ groups and the expression levels of miR-200c-3p were upregulated (**Figure 3D**), suggesting that there may be other mechanisms to regulate DUSP1 or miR-200c-3p expression *via* H_2_O_2_ in the HepG2 cell line. Taken together, the results showed that miR-200c-3p regulated DUSP1 expression at the mRNA level. The following luciferase experiment verified that miR-200c-3p inhibited the expression of DUSP1 by binding the seed sequence of the 3’UTR region (**Figure 3E**).

To further demonstrate our hypothesis, we further verified the results in animal models. The NAFLD+FR groups were included in the animal model to investigate whether a reduction in food intake was sufficient to induce weight loss and metabolic remodeling. Compared with the NAFLD+FR group, the NAFLD+SG group demonstrated greater post-surgical effects on weight loss, liver function enzymology assay, lipid panel (**Figures 4A**–**G**), and pathological phenotypes of NAFLD (**Figure 5A**). In addition, the expression level of miR-200c-3p decreased while DUSP1 increased in the NAFLD+SG group (**Figures 4H, I**).

Last but not the least, the present study validated that SG inactivated the MAPK signaling pathway in NAFLD mice. It has been widely known that activation of ERK can lead to cell proliferation, whereas activation of JNK and p38 causes cell death. The MAPK pathway was an established regulator of hepatic metabolism (42). The interventions of MAPK phosphorylations can protect the liver from inflammations, and inhibitions of the MAPK signaling pathway can improve liver fibrosis (43, 44). The expression levels of p-ERK1/2, p-p38, and p-JNK were significantly downregulated in the NAFLD+SG group compared with the NAFLD+SHAM group and NAFLD+FR group (**Figure 5B**), which was secondary to the upregulation of DUSP1 after SG.

### Limitations

Our study is limited by retrospectively observational design and the short time of following-up, which was based on small sample size. In the cell experiments, only the regulation and direct binding mechanisms between miR-200c-3p and DUSP1 were verified, validation of the MAPK pathway is lacking.

## Conclusion

In conclusion, we reported the inhibition of miR-200c-3p responsible for the overexpression of DUSP1 expression and inactivation of downstream MAPK pathway during NAFLD resolution induced by SG. The study provides meaningful insight into the molecular processes of NAFLD following SG, and that miR-200c-3p may be a therapeutic target in NAFLD pathogenesis.

## Data Availability Statement

The original contributions presented in the study are included in the article/supplementary material. Further inquiries can be directed to the corresponding authors.

## Ethics Statement

The studies involving human participants were reviewed and approved by Ethics Committee of Dalian Municipal Central Hospital. The patients/participants provided their written informed consent to participate in this study. The animal study was reviewed and approved by Ethics Committee of Dalian Municipal Central Hospital.

## Author Contributions

T-tZ was responsible for the collection of samples, statistical analysis, interpretation of data and writing of the manuscript. YW and X-wZ was responsible for the study concept and design, collection of samples, data collection, analysis of dietary records, interpretation of data, editing the manuscript, and study supervision. K-Yy provided help with statistical analysis. X-qM was responsible for the collection of samples. G-hZ was responsible for the interpretation of data and edited the manuscript. All authors approved the final version of the article.

## Funding

Young and middle-aged science and technology innovation talents in Shenyang Support project (RC200607).

## Conflict of Interest

The authors declare that the research was conducted in the absence of any commercial or financial relationships that could be construed as a potential conflict of interest.

## Publisher’s Note

All claims expressed in this article are solely those of the authors and do not necessarily represent those of their affiliated organizations, or those of the publisher, the editors and the reviewers. Any product that may be evaluated in this article, or claim that may be made by its manufacturer, is not guaranteed or endorsed by the publisher.

## References

[1] Cherla DV Rodriguez NA Vangoitsenhoven R Singh T Mehta N McCullough AJ. Impact of Sleeve Gastrectomy and Roux-En-Y Gastric Bypass on Biopsy-Proven non-Alcoholic Fatty Liver Disease. Surg Endosc (2020) 34(5):2266–72. doi: 10.1007/s00464-019-07017-0 31359195

[2] Rinella ME. Nonalcoholic Fatty Liver Disease: A Systematic Review. Jama (2015) 313(22):2263–73. doi: 10.1001/jama.2015.5370 26057287

[3] Diehl AM Day C. Cause, Pathogenesis, and Treatment of Nonalcoholic Steatohepatitis. N Engl J Med (2017) 377(21):2063–72. doi: 10.1056/NEJMra1503519 29166236

[4] Younossi ZM Koenig AB Abdelatif D Fazel Y Henry L Wymer M. Global Epidemiology of Nonalcoholic Fatty Liver Disease-Meta-Analytic Assessment of Prevalence, Incidence, and Outcomes. Hepatology (2016) 64(1):73–84. doi: 10.1002/hep.28431 26707365

[5] Zeng Q Li N Pan X-F Chen L Pan A. Clinical Management and Treatment of Obesity in China. Lancet Diabetes Endocrinol (2021) 9(6):393–405. doi: 10.1016/S2213-8587(21)00047-4 34022157

[6] Murray CJL Aravkin AY Zheng P Abbafati C Abbas KM Abbasi-Kangevari M. Global Burden of 87 Risk Factors in 204 Countries and Territories, 1990–2019: A Systematic Analysis for the Global Burden of Disease Study 2019. Lancet (2020) 396(10258):1223–49. doi: 10.1016/S0140-6736(20)30752-2 PMC7566194 33069327

[7] Li L Liu DW Yan HY Wang ZY Zhao SH Wang B. Obesity is an Independent Risk Factor for non-Alcoholic Fatty Liver Disease: Evidence From a Meta-Analysis of 21 Cohort Studies. Obes Rev (2016) 17(6):510–9. doi: 10.1111/obr.12407 27020692

[8] Chalasani N Younossi Z Lavine JE Diehl AM Brunt EM Cusi K. The Diagnosis and Management of non-Alcoholic Fatty Liver Disease: Practice Guideline by the American Association for the Study of Liver Diseases, American College of Gastroenterology, and the American Gastroenterological Association. Hepatology (2012) 55(6):2005–23. doi: 10.1002/hep.25762 22488764

[9] Asghari S Asghari-Jafarabadi M Somi MH Ghavami SM Rafraf M. Comparison of Calorie-Restricted Diet and Resveratrol Supplementation on Anthropometric Indices, Metabolic Parameters, and Serum Sirtuin-1 Levels in Patients With Nonalcoholic Fatty Liver Disease: A Randomized Controlled Clinical Trial. J Am Coll Nutr (2018) 37(3):223–33. doi: 10.1080/07315724.2017.1392264 29313746

[10] Loomba R Cortez-Pinto H. Exercise and Improvement of NAFLD: Practical Recommendations. J Hepatol (2015) 63(1):10–2. doi: 10.1016/j.jhep.2015.03.009 25863525

[11] Rouabhia S Milic N Abenavoli L. Metformin in the Treatment of non-Alcoholic Fatty Liver Disease: Safety, Efficacy and Mechanism. Expert Rev Gastroenterol Hepatol (2014) 8(4):343–9. doi: 10.1586/17474124.2014.894880 24580044

[12] Sumida Y Yoneda M. Current and Future Pharmacological Therapies for NAFLD/NASH. J Gastroenterol (2018) 53(3):362–76. doi: 10.1007/s00535-017-1415-1 PMC5847174 29247356

[13] American Diabetes Association. Obesity Management for the Treatment of Type 2 Diabetes: Standards of Medical Care in Diabetes-2018. Diabetes Care (2018) 41(Suppl 1):S65–s72. doi: 10.2337/dc18-S007 29222378

[14] Nagarur A Fenves AZ. Late Presentation of Fatal Hyperammonemic Encephalopathy After Roux-En-Y Gastric Bypass. Proc (Baylor Univ Med Center) (2017) 30(1):41–3. doi: 10.1080/08998280.2017.11929521 PMC5242109 28127128

[15] Fenves AZ Shchelochkov OA Mehta A. Hyperammonemic Syndrome After Roux-En-Y Gastric Bypass. Obes (Silver Spring) (2015) 23(4):746–9. doi: 10.1002/oby.21037 25754921

[16] Baltasar A Serra C Pérez N Bou R Bengochea M. Clinical Hepatic Impairment After the Duodenal Switch. Obes Surg (2004) 14(1):77–83. doi: 10.1381/096089204772787338 14980038

[17] Requarth JA Burchard KW Colacchio TA Stukel TA Mott LA Greenberg ER. Long-Term Morbidity Following Jejunoileal Bypass. Continuing Potential Need Surg Reversal Arch Surg (1995) 130(3):318–25. doi: 10.1001/archsurg.1995.01430030088018 7887801

[18] Eilenberg M Langer FB Beer A Trauner M Prager G Staufer K. Significant Liver-Related Morbidity After Bariatric Surgery and Its Reversal-A Case Series. Obes Surg (2018) 28(3):812–9. doi: 10.1007/s11695-017-2925-x PMC5803276 28965313

[19] Sarkhosh K Birch DW Shi X Gill RS Karmali S. The Impact of Sleeve Gastrectomy on Hypertension: A Systematic Review. Obes Surg (2012) 22(5):832–7. doi: 10.1007/s11695-017-2925-x 22350987

[20] Liu Y Shepherd EG Nelin LD. MAPK Phosphatases–Regulating the Immune Response. Nat Rev Immunol (2007) 7(3):202–12. doi: 10.1038/nri2035 17318231

[21] Xanthakos SA Jenkins TM Kleiner DE Boyce TW Mourya R Karns R. High Prevalence of Nonalcoholic Fatty Liver Disease in Adolescents Undergoing Bariatric Surgery. Gastroenterology (2015) 149(3):623–34.e8. doi: 10.1053/j.gastro.2015.05.039 26026390 PMC4654456

[22] Kornfeld JW Baitzel C Könner AC Nicholls HT Vogt MC Herrmanns K. Obesity-Induced Overexpression of miR-802 Impairs Glucose Metabolism Through Silencing of Hnf1b. Nature (2013) 494(7435):111–5. doi: 10.1038/nature11793 23389544

[23] Lee Y Jeon K Lee J-T Kim S Kim VN. MicroRNA Maturation: Stepwise Processing and Subcellular Localization. EMBO J (2002) 21(17):4663–70. doi: 10.1093/emboj/cdf476 PMC126204 12198168

[24] Lee Y Ahn C Han J Choi H Kim J Yim J. The Nuclear RNase III Drosha Initiates microRNA Processing. Nature (2003) 425(6956):415–9. doi: 10.1038/nature01957 14508493

[25] Hutvágner G Zamore PD. A microRNA in a Multiple-Turnover RNAi Enzyme Complex. Sci (New York NY) (2002) 297(5589):2056–60. doi: 10.1126/science.1073827 12154197

[26] Zhu M Wang Q Zhou W Liu T Yang L Zheng P. Integrated Analysis of Hepatic mRNA and miRNA Profiles Identified Molecular Networks and Potential Biomarkers of NAFLD. Sci Rep (2018) 8(1):7628. doi: 10.1038/s41598-018-25743-8 29769539 PMC5955949

[27] Fan JG Jia JD Li YM Wang BY Lu LG Shi JP. Guidelines for the Diagnosis and Management of Nonalcoholic Fatty Liver Disease: Update 2010: (Published in Chinese on Chinese Journal of Hepatology 2010; 18:163-166). J Dig Dis (2011) 12(1):38–44. doi: 10.1111/j.1751-2980.2010.00476.x 21276207

[28] Harrison SA Fecht W Brunt EM Neuschwander-Tetri BA. Orlistat for Overweight Subjects With Nonalcoholic Steatohepatitis: A Randomized, Prospective Trial. Hepatology (2009) 49(1):80–6. doi: 10.1002/hep.22575 19053049

[29] Promrat K Kleiner DE Niemeier HM Jackvony E Kearns M Wands JR. Randomized Controlled Trial Testing the Effects of Weight Loss on Nonalcoholic Steatohepatitis. Hepatology (2010) 51(1):121–9. doi: 10.1002/hep.23276 PMC2799538 19827166

[30] Gloy VL Briel M Bhatt DL Kashyap SR Schauer PR Mingrone G. Bariatric Surgery Versus non-Surgical Treatment for Obesity: A Systematic Review and Meta-Analysis of Randomised Controlled Trials. BMJ (Clinical Res ed) (2013) 347:f5934. doi: 10.1136/bmj.f5934 PMC3806364 24149519

[31] Chavez-Tapia NC Tellez-Avila FI Barrientos-Gutierrez T Mendez-Sanchez N Lizardi-Cervera J Uribe M. Bariatric Surgery for non-Alcoholic Steatohepatitis in Obese Patients. Cochrane Database Syst Rev (2010) 2010(1):Cd007340. doi: 10.1002/14651858.CD007340.pub2 PMC7208314 20091629

[32] European Association for Study of Liver Asociacion Latinoamericana para el Estudio del Higado. EASL-ALEH Clinical Practice Guidelines: Non-Invasive Tests for Evaluation of Liver Disease Severity and Prognosis. J Hepatol (2015) 63(1):237–64. doi: 10.1016/j.jhep.2015.04.006 25911335

[33] Esquivel CM Garcia M Armando L Ortiz G Lascano FM Foscarini JM. Laparoscopic Sleeve Gastrectomy Resolves NAFLD: Another Formal Indication for Bariatric Surgery? Obes Surg (2018) 28(12):4022–33. doi: 10.1007/s11695-018-3466-7 30121855

[34] Feng YY Xu XQ Ji CB Shi CM Guo XR Fu JF. Aberrant Hepatic microRNA Expression in Nonalcoholic Fatty Liver Disease. Cell Physiol Biochem (2014) 34(6):1983–97. doi: 10.1159/000366394 25562147

[35] Dou L Zhao T Wang L Huang X Jiao J Gao D. miR-200s Contribute to Interleukin-6 (IL-6)-Induced Insulin Resistance in Hepatocytes. J Biol Chem (2013) 288(31):22596–606. doi: 10.1074/jbc.M112.423145 PMC3829346 23798681

[36] Ramachandran S Ilias Basha H Sarma NJ Lin Y Crippin JS Chapman WC. Hepatitis C Virus Induced Mir200c Down Modulates FAP-1, a Negative Regulator of Src Signaling and Promotes Hepatic Fibrosis. PloS One (2013) 8(8):e70744. doi: 10.1371/journal.pone.0070744 23950995 PMC3741284

[37] Wu JJ Roth RJ Anderson EJ Hong EG Lee MK Choi CS. Mice Lacking MAP Kinase Phosphatase-1 Have Enhanced MAP Kinase Activity and Resistance to Diet-Induced Obesity. Cell Metab (2006) 4(1):61–73. doi: 10.1016/j.cmet.2006.05.010 16814733

[38] Lawan A Zhang L Gatzke F Min K Jurczak MJ Al-Mutairi M. Hepatic Mitogen-Activated Protein Kinase Phosphatase 1 Selectively Regulates Glucose Metabolism and Energy Homeostasis. Mol Cell Biol (2015) 35(1):26–40. doi: 10.1128/MCB.00503-14 25312648 PMC4295383

[39] Singh GB Raut SK Khanna S Kumar A Sharma S Prasad R. MicroRNA-200c Modulates DUSP-1 Expression in Diabetes-Induced Cardiac Hypertrophy. Mol Cell Biochem (2017) 424(1-2):1–11. doi: 10.1007/s11010-016-2838-3 27696308

[40] Petiz LL Kunzler A Bortolin RC Gasparotto J Matté C Moreira JCF. Role of Vitamin A Oral Supplementation on Oxidative Stress and Inflammatory Response in the Liver of Trained Rats. Appl Physiol Nutr Metab (2017) 42(11):1192–200. doi: 10.1139/apnm-2017-0193 28742973

[41] Zhou JY Liu Y Wu GS. The Role of Mitogen-Activated Protein Kinase Phosphatase-1 in Oxidative Damage-Induced Cell Death. Cancer Res (2006) 66(9):4888–94. doi: 10.1158/0008-5472.CAN-05-4229 16651445

[42] Johnson GL Lapadat R. Mitogen-Activated Protein Kinase Pathways Mediated by ERK, JNK, and P38 Protein Kinases. Sci (New York NY) (2002) 298(5600):1911–2. doi: 10.1126/science.1072682 12471242

[43] Lawan A Bennett AM. Mitogen-Activated Protein Kinase Regulation in Hepatic Metabolism. Trends Endocrinol Metab (2017) 28(12):868–78. doi: 10.1016/j.tem.2017.10.007 PMC5774993 29128158

[44] Jiang J Yan L Shi Z Wang L Shan L Efferth T. Hepatoprotective and Anti-Inflammatory Effects of Total Flavonoids of Qu Zhi Ke (Peel of Citrus Changshan-Huyou) on non-Alcoholic Fatty Liver Disease in Rats *via* Modulation of NF-κb and MAPKs. Phytomedicine (2019) 64:153082. doi: 10.1016/j.phymed.2019.153082 31541796

